# When Flow Creates Order: Nematic Alignment and Form Birefringence in Shear for High-Density Polyethylene Melts

**DOI:** 10.1021/acs.macromol.6c00994

**Published:** 2026-07-20

**Authors:** Arshiya Bhadu, Alicyn M. Rhoades, Ralph H. Colby

**Affiliations:** † Department of Materials Science and Engineering, Penn State University, University Park, Pennsylvania 16802, United States; ‡ School of Engineering, 43312 Penn State Behrend, Erie, Pennsylvania 16563, United States

## Abstract

Making use of the exponential character of the high molecular weight tail of high-density polyethylene allows the quantification of the fraction of chains that get stretched in shear flow, which tracks with measured birefringence and Raman intensity. At high enough shear rates, apparent viscosity and birefringence show the shear rate dependence of chains that exhibit nematic alignment. In the nematic regime, a strong increase in birefringence and simultaneous strong decrease in primary normal stress difference mean there is a failure of the linear stress-optical rule, suggesting that form birefringence results from nematic alignment, which was confirmed using optical techniques.

## Graphical abstract

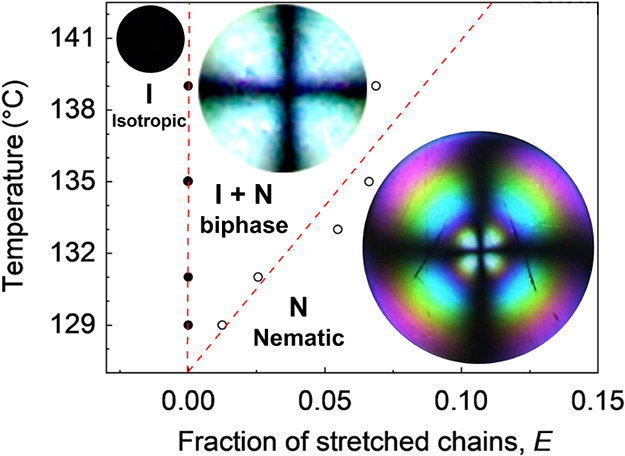

## Introduction

An interval of shear flow applied to a polymer melt prior to crystallization can strongly accelerate crystallization kinetics. Janeschitz-Kreigl^1^ proposed that the control parameter for such flow-induced crystallization (FIC) seems to be specific work *W*. At a constant shear rate γ̇, the applied specific work^1–8^ *W* = γ̇∫_0_^*t*_*s*_^σd*t* (page 141 of ref 1), where σ is the shear stress and *t*_*s*_ is shearing time. This control parameter has been tested for a variety of semicrystalline polymers, although usually only over quite limited ranges of shear rate. The shear rate needs to be larger than some critical value to get any FIC effect, while edge fracture in rotational rheometers limits how high shear rates can be studied. Our group has studied FIC in detail and concluded that *W* is the control parameter for isotactic polypropylenes (iPP),^2,3,5,6,9^ polyamide 6,6 ^7^ and poly­(ether ether ketone) (PEEK);^4,7,10,11^ others have reached the same conclusion for high-density polyethylene (HDPE).^12^ *W* only fails to be the control parameter for FIC^13^ when very different flows are used to reach the same level of *W*. To generate the same *W* for an iPP sample, Jacob et al.^13^ used 0.2 s^–1^ for 4128 s and 98.6 s^–1^ for 1 s and observed that the smaller shear rate resulted in considerably less anisotropy in SAXS at ambient temperature.

The underlying thermodynamics were proposed long ago by Flory,^14^ using the idea that any flow that stretches some chains lowers the configurational entropy (elastocaloric effect), with entropy reduction Δ*S* < 0. At a given shearing temperature *T*_*S*_, the reduction in configurational entropy increases the free energy of the sheared melt by −*T*_*S*_Δ*S*. Since the *W* applied to the system is always higher than −*T*_*S*_Δ*S* due to energy losses such as heat dissipation, the energy related to the fraction of chains in the melt that were actually stretched by the flow can be expressed using the energy conversion factor^4^ (*E*), with *EW* = −*T*_*S*_Δ*S*.

Only the longest chains in the molecular weight distribution get stretched in shear flow,^3,15^ as the shear rate needs to exceed the reciprocal of their Rouse relaxation time (τ_*R*_ ∼ *N* ^2^). For this reason, when the concept of the energy conversion factor was first proposed for polydisperse PEEK melts at modest shear rates, *E* was found to be 0.01. This means that only 1 wt % of the PEEK chains, presumably in the high molecular weight tail of the distribution, got stretched and lowered configurational entropy, while the other 99% of chains dissipate the applied shear energy as heat.

Similarly for HDPE, the high molecular weight tail in its molecular weight distribution can be used to estimate the fraction of chains that stretch under shear, which correlates with birefringence and Raman signals. At high shear rates, HDPE exhibits behavior consistent with nematic alignment, reflected in changes in viscosity and a pronounced increase in birefringence. In this regime, the breakdown of the linear stress–optical rule indicates that birefringence is dominated by nematic ordering, as confirmed by optical measurements.

## Results and Discussion

### Proposed Model for Chain Stretching Using the Molecular Weight Distribution

Commercial polymers are polydisperse and there is no particular longest chain that can be resolved in any real molecular weight distribution. Long chains are simply exponentially rare, with most distributions having an exponential cutoff in the long chain limit. The large *N* asymptotic form of the most-probable weight fraction distribution for linear polycondensation polymers is approximated via a linear function with an exponential cutoff, $w_{N}\approx \frac{N}{N_{n}^{2}}\exp\left(\frac{-N}{N_{n}}\right)$ (eq 1.65 of ref 16) and the Shultz distribution (with an additional adjustable parameter) approximates the weight fraction for linear addition polymers, $w_{N}=\frac{1}{N_{n}\Gamma \left(s\right)}{\left(\frac{sN}{N_{n}}\right)}^{s}\exp\left(\frac{-sN}{N_{n}}\right)$ (eq 1.70 of ref 16). With six iPP samples having very different molecular weight distributions composed of only linear chains, the tail of each weight fraction distribution *w*_*N*_ has been successfully fit to an exponential cutoff.^3^

$$w_{N}=\frac{1}{N^{*}}\exp\left(\frac{-N}{N^{*}}\right)$$ 1

Here *N* is the number of Kuhn monomers in a given chain and N* controls the exponential cutoff. An example is shown in Figure 1 using gel permeation chromatography (GPC) data for the HDPE sample used herein from Sigma-Aldrich (product no. 547999). Fitting the data for *N* > 2000 in Figure 1b concludes that N* = 4400, allowing quantification of the weight fractions of any chain in the distribution with more than 2000 Kuhn monomers.

**Figure 1:**
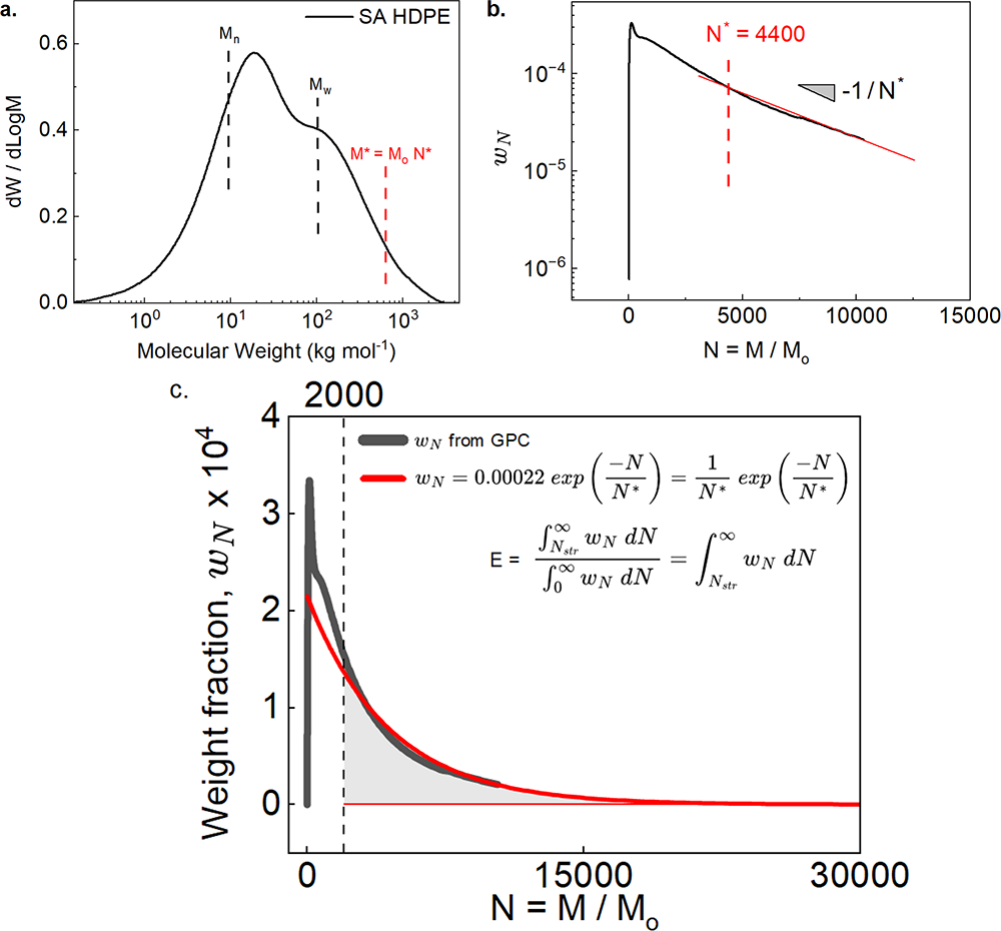
Molar mass distribution of HDPE studied in this paper. (a) Molecular weight distribution of HDPE in the standard form used by GPC experts where the dispersity (*M*_w_/*M*_n_) is 10.4. (b) Semilog fitting procedure for N*, which is obtained by plotting the logarithm of the weight fraction versus linear number of Kuhn monomers per chain *N*, where the slope at large *N* is defined as −1/N* from eq 1. (c) Experimental weight fraction of HDPE (black line) as a function of *N*, where the tail of the distribution starting from *N* = 2000 is well described using eq 1 (red line). The integral of the weight fraction from *N*_*str*_ to *∞* (gray shaded area) determines the fraction of chains that get stretched *E*.

Our hypothesis is that the energy conversion factor *E* is the weight fraction of chains that get stretched at a given shear rate (set by their Rouse time^3,15,17^ ). We define *N*_str_ as the shortest chains that get stretched at a given shear rate and integrate the weight fraction distribution (eq 1) over all chains larger than *N*_str_ (gray area in Figure 1c) to obtain the fraction of chains that get stretched *E*.

$$E=\frac{\int_{N_{\mathrm{str}}}^{\infty }w_{N}dN}{\int_{0}^{\infty }w_{N}dN}=\exp\left(\frac{-N_{\mathrm{str}}}{N^{*}}\right)$$ 2

Figure 1c compares the experimental weight fraction of HDPE from GPC with the weight fraction expected by eq 1, which nicely fits the tail of the distribution. Fitting the linear viscoelastic response^18,19^ of HDPE to the BoB model^5,20^ (with the input being molecular weight distribution) determines the Rouse time of an entanglement strand τ_*e*_ at any given temperature. HDPE has *N*_*e*_ = 6.9 Kuhn monomers in an entanglement strand^21,22^ (the molar mass of a Kuhn monomer for HDPE is *M*_o_ = 0.150 kg mol^–1^)^16^ and τ_*e*_ = 6.4 ns at 129 °C. Then the Rouse time of any chain of *N* Kuhn monomers can be calculated as τ_*R*_(*N*) = τ_*e*_(*N*/*N*_*e*_)^2^ = τ_*o*_ *N* ^2^, where τ_*o*_ = 0.134 ns is the Rouse time of the Kuhn monomer (shortest Rouse time^16^) at 129 °C. This leads to the shear rate dependence of the critical number of Kuhn monomers in the shortest chains that get stretched at a given shear rate (*N*_str_).

$$N_{\mathrm{str}}=\sqrt{\frac{\tau_{R,\mathrm{str}}}{\tau_{o}}}=\sqrt{\frac{1}{\mathrm{\gamma ̇}\tau_{o}}}$$ 3

As the shear rate is increased, *N*_*str*_ decreases, meaning more chains get stretched. Combining eqs 2 and 3 yields the shear rate dependence of *E*.

$$E=\exp\left(\frac{-1}{N^{*}\sqrt{\dot{\gamma }\tau_{o}}}\right)\;\mathrm{for}\;N>2000$$ 4

*E* ranges from 0 to 1 (Figure 2a), and is directly equal to the weight fraction of chains that are long enough to stretch at a particular shear rate.

**Figure 2:**
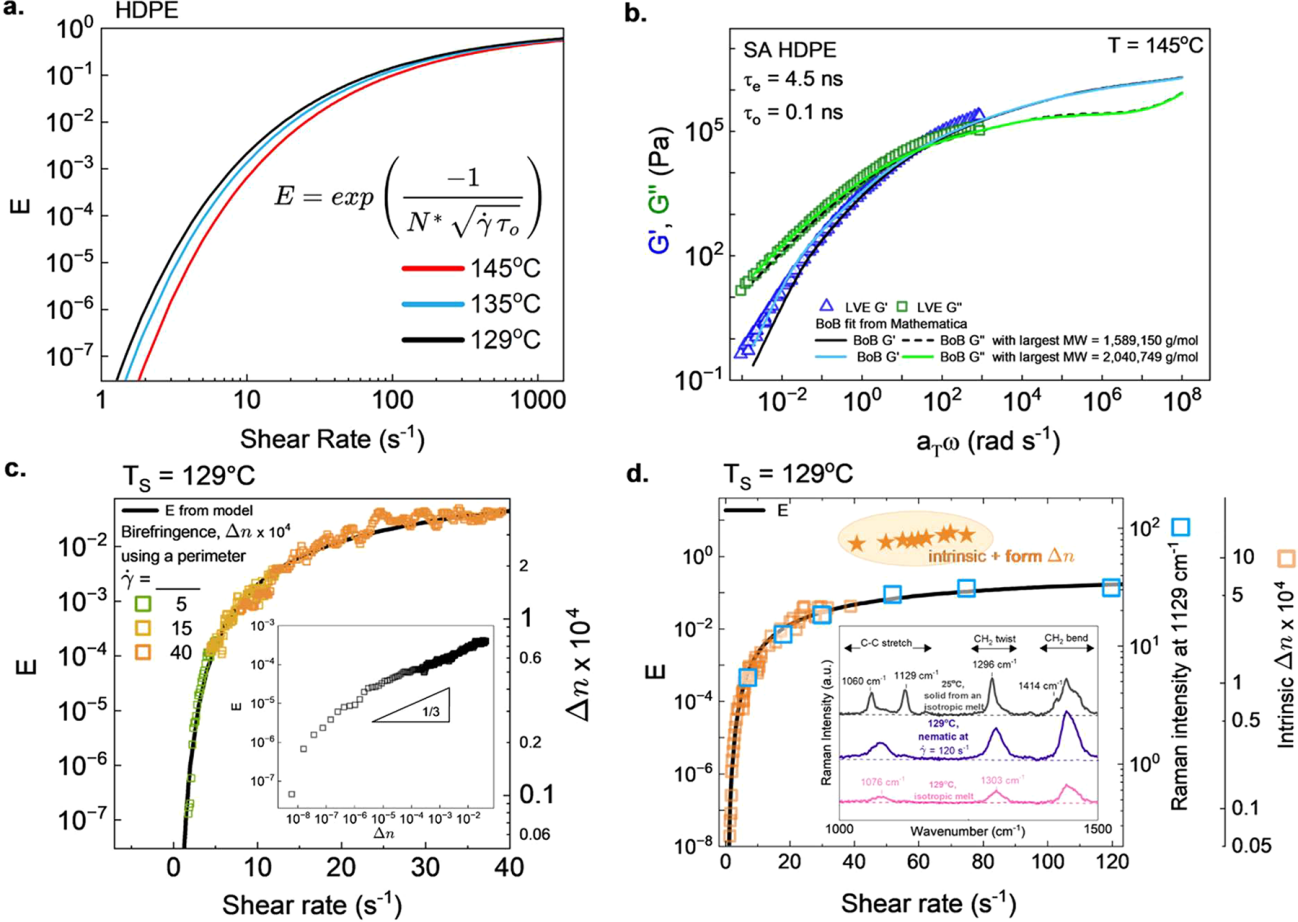
(a) Energy conversion factor of HDPE as a function of shear rate using eq 4 at three temperatures. (b) BoB fit can only resolve the longest chains in HDPE up to 10,000 Kuhn monomers. The BoB fit based on the GPC data (black curves) does not work below 0.1 rad s^–1^ because of chains longer than 1,500 kg mol^–1^ that the GPC cannot resolve. Inputting the exponential tail of eq 1 (Table 1 in the Supporting Information) enables an excellent BoB fit (light blue and light green curves for G′ and G″, respectively). (c) Birefringence follows the increase in *E* as shear rate increases starting from *E* = 10^–7^, corresponding to *N*_str_ = 50,000. Light intensity was measured using an in situ shear induced polarized light imaging setup (SIPLI)^10,11,23–25^ equipped with a red light source (633 nm). Birefringence is used to calibrate the light intensity data shown here (see Supporting Information for more details). While Raman intensity is proportional to *E*, Δ*n* ∼ E^1/3^. (d) HDPE was sheared at a wide range of shear rates at 129 °C (new sample for each shear rate) and studied via a Raman probe, where along with the peaks associated with the HDPE melt at 1076 and 1303 cm^–1^, there is a peak detected at 1129 cm^–1^, which represents a conformation with consecutive trans ethylene repeat units.^26–29^ The inset shows the Raman spectra for an isotropic HDPE melt at 129 °C (pink curve), a nematic HDPE at 129 °C using a shear rate of 120 s^–1^ (purple curve), and semicrystalline HDPE at 25 °C (dark gray curve).

### Experimental Validation Using Birefringence and Raman Spectroscopy

Figure 2a plots the predicted shear rate dependence of *E* for our HDPE sample at three temperatures. Note that we are using eq 4 much farther into the high molecular weight tail than any GPC experiment can resolve. The longest chains that can be clearly resolved above the baseline in Figure 1 have *N* = 10,000 Kuhn monomers (*M* = 1500 kg mol^–1^). If we take *N*_str_ = 10,000 then *E* = exp­(−10,000/4400) = 0.103. It is the longest chains in the molecular weight distribution that determine the low frequency linear viscoelastic (LVE) response of a polymer. If the BoB fitting had been performed with the longest chain having a 1589 kg mol^–1^ molecular weight, the BoB fit fails to capture G’ for ω < 0.1 rad s^–1^ as shown by the black curves in Figure 2b. Making use of the fit to eq 1 concludes that there is a weight fraction 0.005 of chains with *M* = 2000 kg mol^–1^ and including those results in a very good BoB fit (Figure 2b). Figure 2c compares the predicted shear rate dependence of *E* with the magnitude of measured birefringence, quantified by the intensity of light (633 nm wavelength) inside the first fringe. The smallest shear rate with measurable birefringence (γ̇ = 3 s^–1^) in Figure 2c has *E* = 10^–7^, corresponding to *N*_st*r*_ = 50,000, i.e., a molecular weight of 7500 kg mol^–1^, far beyond the resolution of any GPC.

The observation that the magnitude of the birefringence tracks nicely with *E* not only gives us confidence in our calculations but also has a strong significance in polymer physics. The birefringence measured must be from stretched longer chains coexisting with partly oriented shorter chains that dissipate their portion of the applied specific work as heat. This is consistent with de Gennes’ idea^30^ (proposed in 1974 for dilute solutions) that there is a first order coil–stretch transition; stretched and unstretched chains coexist.

Nematic alignment due to chain stretching is further supported by in situ Raman spectroscopy of the HDPE melt at *T*_*S*_ = 129 °C. There is a peak at 1129 cm^–1^ in the melt during shear at 129 °C, which suggests there is a small population of strongly stretched chains, as this peak corresponds to a conformation with multiple consecutive trans ethylene^26–29^ repeat units. The increase in Raman intensity at 1129 cm^–1^ as a function of shear rate follows the *E* model much like birefringence, as shown in Figure 2d, and this provides valuable insight about the conformation of HDPE during nematic alignment. The appearance of the 1129 cm^–1^ peak is significant as it is indistinguishable in isotropic HDPE. The peak is small because the fraction of long chains that stretch is small. Below 20 s^–1^ in the biphase regime, the polyethylene chains have less than ten consecutive trans ethylene repeat units^26^ (low intensity peak at 1129 cm^–1^). Above 40 s^–1^, in the nematic regime, the polyethylene chains reach a larger number of consecutive trans ethylene repeat units^26^ as a function of shear rate, suggesting strong nematic alignment.

Short polyethylene chains exhibit a different metastable precursor to the crystalline phase known as the rotator phase.^31–33^ In the rotator phase, polyethylene chains are less ordered compared to the crystalline phase, and are free to rotate around their aligned long axis.^31^ The nematic phase exhibits strong alignment and no signals of being a rotator phase precursor to the crystalline phase, as there is no peak at 1296 cm^–1^ expected for the rotator phase.^32,33^ Monte Carlo simulations by Kawak^34^ show that crystallization in polymers, even quiescently, is preceded by the formation of an intermediary nematic phase, consistent with our results. During the formation of consecutive all-trans ethylene units, a peak at 1060 cm^–1^ is also prevalent, though it is weak in the HDPE samples. Since Raman signals are weaker in polymer melts compared to birefringence, the lowest shear rate where the 1129 cm^–1^ peak is observed was 7 s^–1^ (where *E* = 6 × 10^–4^) at 129 °C, while birefringence is a more sensitive measurement of chain stretching even at 2 s^–1^ (where *E* = 10^–7^) at 129 °C. The intrinsic birefringence values follow the *E* curve, and the jump in birefringence above 40 s^–1^ (star symbols in Figure 2d) quantifies the portion of birefringence that is due to form effects at the higher shear rates. The form birefringence will be discussed in detail below.

### Flow-Induced Nematic Alignment in HDPE Because of Long Chain Stretching

PEEK and HDPE both show a nematic alignment of chains in sufficiently strong shear flow. Zhang and Larson^35,36^ hypothesize that at *T* < 300 K a nematic phase is a metastable precursor to the crystalline phase in a polyethylene melt using atomistic simulation and self-consistent field theory, by quenching pentacontane (C50) to below its I–N transition temperature (≈ 300 K). Recently, Zhang reported that the nematic transforms into a rotator phase^32^ around 350 K, which then acts as the precursor to the crystalline phase in a polyethylene melt. This suggests that HDPE melts at *T*_m_ are not very far above their equilibrium nematic-to-isotropic transition temperature. Undercooling to 300–350 K is not possible experimentally, as HDPE always crystallizes^37^ too fast. Here we instead increase γ̇ to significantly reduce the configurational entropy of long chains by stretching them to stabilize the nematic alignment of the longest chains in shear flow at higher temperatures.

With PEEK, there is a strong failure of the Cox–Merz rule,^10,11^ which started at the shear rate where strong birefringence is first seen, with high shear rate viscosity scaling as the reciprocal square root of shear rate, and was interpreted as strong evidence of a flow-induced nematic alignment of long chains. Figure 3 shows that a very similar strong failure of the Cox–Merz rule,^18^ with high shear rate viscosity scaling as the reciprocal square root of shear rate, is seen for HDPE at 129 °C.

**Figure 3:**
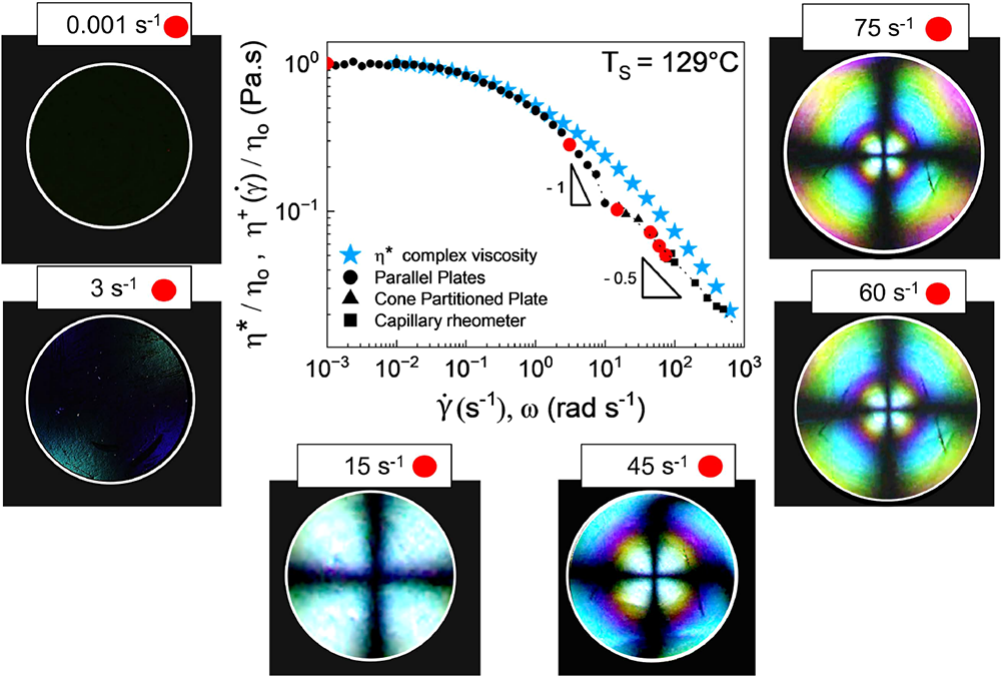
Flow Curve of HDPE at 129 °C exhibiting a shear-induced isotropic to nematic transition. Colors are from light retardance and enable birefringence to be quantified using a white light source. The red filled circles on the flow curve depict the shear rates corresponding to the 2D-SIPLI images, where each image is from separate start-up of steady shear experiments. The SIPLI uses a parallel plate geometry with shear rate linear in radial position. Each color transition determines birefringence at a particular shear rate. The videos of two of the shearing experiments (perimeter shear rates of 45 and 75 s^–1^) are shown in Supporting Information in both real time (1× speed) and quarter time (0.25× speed) for clarity.

The birefringence starts to be measurable at roughly 3 s^–1^, which is also the critical shear rate below which this HDPE shows no FIC (exactly as was observed for PEEK^4,10,11^ ). Above 3 s^–1^, there is a very strong failure of the Cox–Merz rule as the shear viscosity becomes smaller than the oscillatory shear complex viscosity, presumably resulting from more chains getting stretched as shear rate is increased, since stretched chains abandon their entanglements. For the high shear rates above 20 s^–1^, we see the Marrucci slope^17,38–42^ of −1/2 that is characteristic of all nematic polymers.

Another characteristic of nematic polymers is that birefringence scales as the square root of shear rate^43–47^ and Figure 4a shows that we observe that for HDPE in the range 20–80 s^–1^. The birefringence color transitions observed at a given radial position in the 2D-SIPLI images is contrasted with the light path difference in the Michel-Lévy chart to quantify the birefringence^48^ with sample thickness twice the measuring gap. Two different sample thicknesses (0.7 and 1.4 mm) were used to determine the path difference^23^ for the various shear rates. Examples using perimeter shear rates of 45 and 75 s^–1^ are shown in Figure 4. A larger thickness leads to transmission of higher order colors which makes it easier to quantify path difference, as opposed to very small thicknesses which only transmit white light inside the first fringe for the lower shear rates. Using perimeter shear rates of 45 and 75 s^–1^, the birefringence values were evaluated using fresh sample loadings and as expected, regardless of the path difference and sample thickness, the birefringence is only a function of shear rate as shown in Figure 4a.

**Figure 4:**
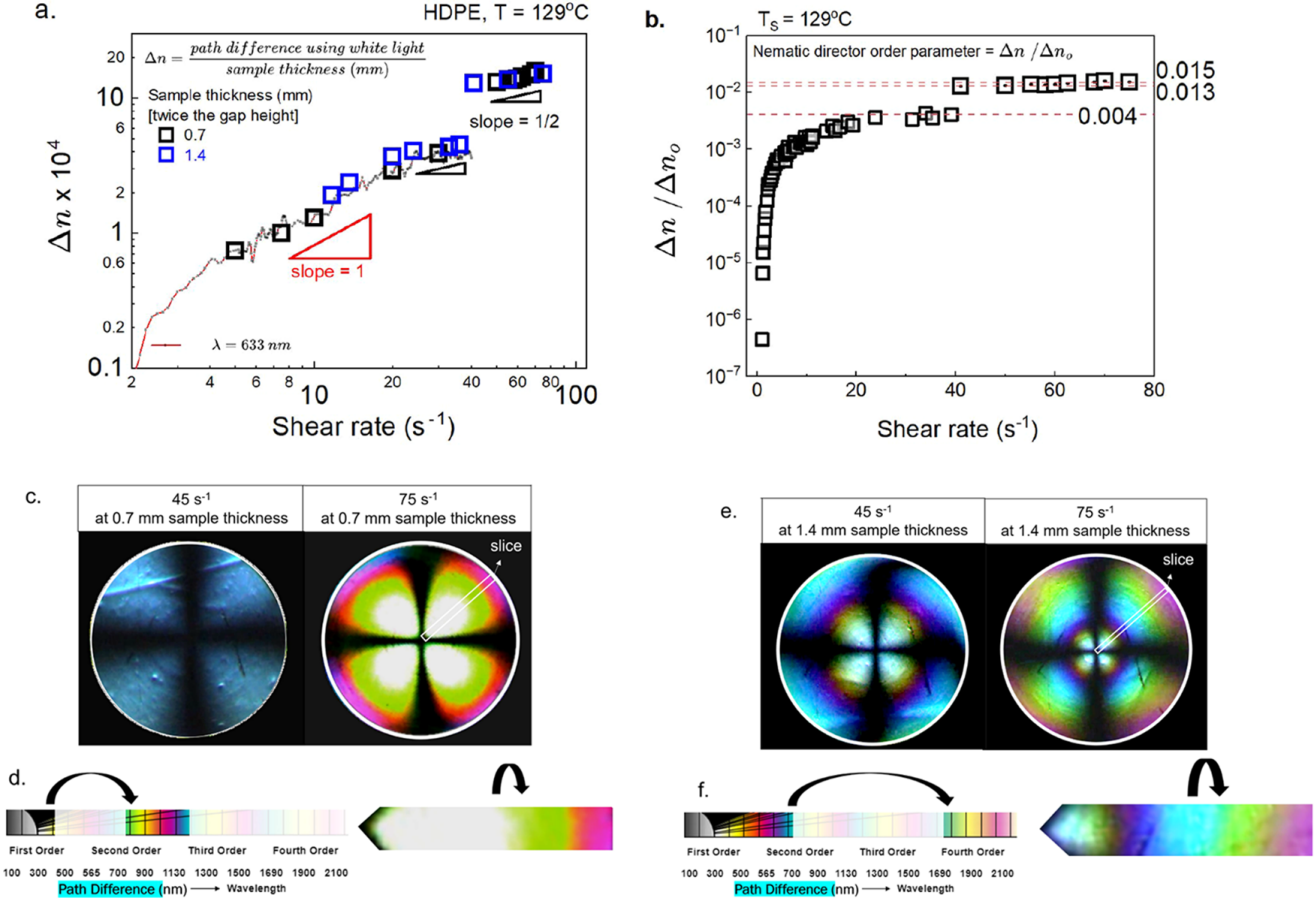
(a) Birefringence, Δ*n*, of HDPE increases with shear rate. Once the nematic forms in the 20–45 s^–1^ and also 45–80 s^–1^ range we see the slope of 1/2 that is characteristic of all nematic polymers^43–47^ under shear flow. Δ*n* was evaluated at various radii of the 2D-SIPLI images as a function of shear rate using the Michel-Lévy chart to determine path difference. (b) Using the intrinsic birefringence^49^ of HDPE (Δ*n*_*o*_ = 0.1 at 129 °C), the nematic director order parameter^50^ can be quantified as Δ*n*/Δ*n*_*o*_. When birefringence makes a sudden increase, Δ*n*/Δ*n*_*o*_ = 0.013 for the sheared HDPE melt. Two different sample thicknesses (0.7 and 1.4 mm) were used to determine the path difference for the various shear rates using perimeter shear rates of 45 and 75 s^–1^ and were utilized to calibrate the light intensity using a 633 nm laser light source. Light retardance at 0.7 mm sample thickness is shown in (c), where (d) shows the respective path difference in the Michel-Lévy chart^51^ for a slice of the SIPLI image at a 45° angle between the Maltese crosses, with a perimeter shear rate of 75 s^–1^. Similarly, light retardance at 1.4 mm sample thickness is shown in (e), where (f) shows the 45° slice. A jump in path difference occurs in the nematic regime from white to a green color at 0.7 mm sample thickness and from cyan to a light green color at 1.4 mm sample thickness.

There is a factor of 2.8 times sudden increase in birefringence in the nematic regime above 40 s^–1^ because nematic alignment is accompanied by *form birefringence* ^15,52^ that increases the total birefringence of HDPE as we will show in the next section. This strong increase in birefringence was observed for PEEK as well^11^ but was not discussed further since it was not understood at the time. The intrinsic birefringence^49^ of HDPE Δ*n*_*o*_ = $5C\rho RT/M_{0}$ = 0.1 at 129 °C, where ρ is mass density and *M*_0_ = 0.150 kg mol^–1^ is the molecular weight of a Kuhn monomer. Δ*n*_*o*_ can be used to evaluate the nematic director order parameter^50^ as Δ*n*/Δ*n*_*o*_. Before the sudden increase in birefringence, Δ*n*/Δ*n*_*o*_ = 0.004. When the birefringence makes a sudden increase, suddenly Δ*n*/Δ*n*_*o*_ = 0.013 which increases with increasing shear rate up to 0.015 at the highest shear rate studied (75 s^–1^).

### Failure of the Stress-Optical Rule in the Nematic Regime Is Accompanied by Form Birefringence

The birefringence can also be estimated using the stress-optical rule that correlates Δ*n* with the primary (*N*_1_) and secondary (*N*_2_) normal stress differences, Δ*n* = *C*(*N*_1_ + *N*_2_) in parallel plate geometry, where C is the stress-optical coefficient.^25,53^ Assuming that *N*_2_ is negligible compared to *N*_1_, Δ*n* ≅ *CN*_1_ where the mean stress-optical coefficient of polyethylene using literature estimates^53,54^ is *C̅* = 1.74 × 10^–9^ Pa^–1^. Using the Δ*n* values from Figure 4 and the *N*_1_ values from rheological measurements, *C* = Δ*n*/*N*_1_ is evaluated over a wide range of shear rates (Figure 5a) using steady-state values of *N*_1_ (Figure 5b) from 8 mm cone and plate and 6 mm cone-partitioned plate^55^ (CPP) geometries in the steady shear start-up^18^ experiment.

**Figure 5:**
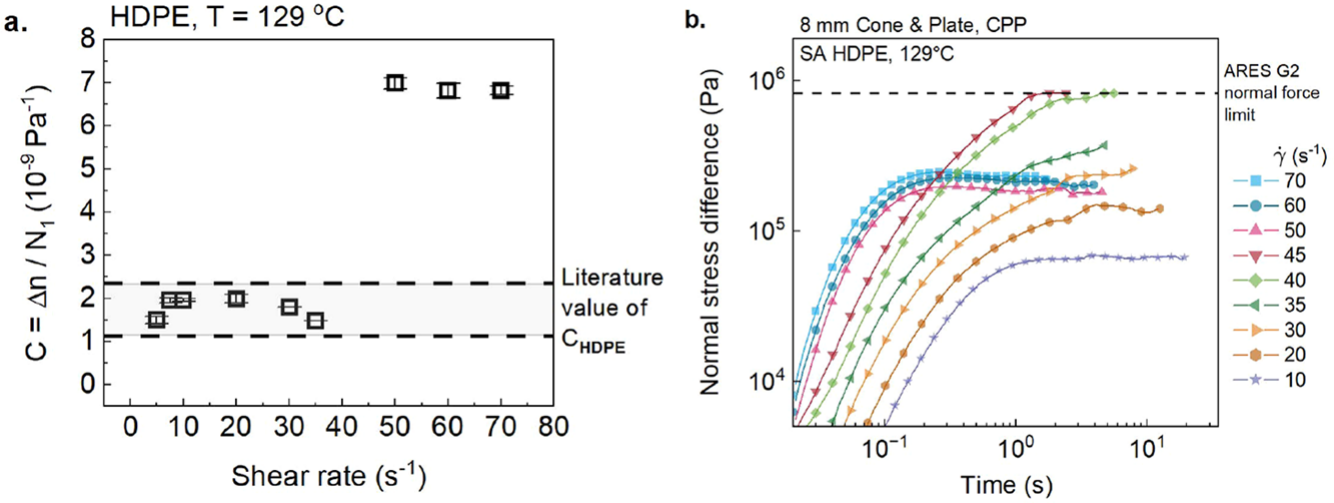
Linear stress-optical rule fails in the fully nematic regime above 45 s^–1^ and is accompanied by a strong decrease in normal stress difference. (a) Stress-optical coefficient (C) of HDPE as a function of shear rate using *N*_1_ from rheology (8 mm cone and plate and 6 mm CPP) where Δn is from Figure 4. (b) Transient normal stress difference *N*_1_ during shear start-up at nine shear rates. Due to the rheometer normal force overload limits, *N*_1_ at 40 and 45 s^–1^ could unfortunately not be measured. Above 45 s^–1^ the normal stresses drop due to stronger nematic alignment of HDPE chains, and the normal stresses can then be measured and reach steady state within 1 s.

The stress-optical rule works (*C* matches literature range^53,54^ ) up to 40 s^–1^ as only a small subset of the sample has nematic alignment, and the transmitted birefringence is a result of the stress that stretches the small fraction of long chains. But as the shear rate increases to above 40 s^–1^ and enough chains get stretched, the melt exhibits a 5 times higher *C* = 7 × 10^–9^ Pa^–1^ that corresponds to nematic alignment in polyethylene (Figure 5a). Since the birefringence only jumped by 2.8 times above 40 s^–1^, this larger increase in *C* is due to the 1.7 times *decrease* in *N*_1_ that occurred upon nematic alignment in HDPE as shown in Figure 5b. The stress-optical rule failing above 40 s^–1^ is a characteristic of form birefringence.^52,56,57^ The molecular dynamics simulation studies on linear polyethylene melts also observed a failure of the stress-optical rule after the intermediate shear flow regime,^58,59^ but they could not explain the reason why, and we believe this is because the simulations did not consider nematic alignment that leads to form birefringence.

The source of birefringence in stretched polymers has been assumed in the literature to be intrinsic,^53^ as the linear stress-optical rule has worked for most polymers. Here we show that there is also another source of birefringence known as form birefringence, which likely occurs because of bundling of the HDPE chains during nematic alignment. Using a half-wave plate (λ/2) in the optical train after the polarizer can confirm the form birefringence as shown in Figure 6. The λ/2 plate rotates the polarization by 90° when oriented at 45° to the incident polarization plane. When intrinsic birefringence occurs (valid linear stress-optical rule), the λ/2 plate systematically inverts the polarization state of the transmitted light. This geometrically counters the uniform retardation introduced by the sample relative to the analyzer, leading to a dark image. The structural anisotropy of nematic HDPE creates form birefringence which produces an additional retardation that is not accounted for by the λ/2 plate, allowing light to pass through the crossed analyzer. Therefore, if birefringence is detected for a polymer melt using the λ/2 plate,^100,101^ it can only arise because of a form effect. Form birefringence could be caused by Mie scattering from an anisotropic object, such as a cylinder formed from stretched chains.

**Figure 6:**
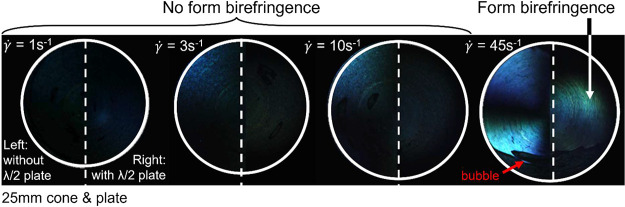
Validating the emergence of form birefringence using a 25 mm Cone and Plate geometry. Form birefringence occurs only at 45 s^–1^ (and higher, not shown) where the stress-optical rule failed as shown using a λ/2 plate. The left side of the SIPLI images, separated with the dashed white line, is without the λ/2 plate, and the right side of the SIPLI images is with the λ/2 plate. The birefringence seen at lower shear rates is only due to intrinsic effects, as the sample is fully dark on the right side when a λ/2 plate is added to the optical train of SIPLI.

At 1 s^–1^, no chain stretch occurs and hence no birefringence can be detected with or without the λ/2 plate as shown in Figure 6b. Here a 25 mm cone and plate geometry was utilized to ensure that the shear rate remains constant throughout the sample area and to make it easier to investigate form effects at various shear rates. Since the sample thickness in a 25 mm cone and plate geometry increases as the radius of the sample area increases, the light intensity should be slightly stronger along the perimeter where the thickness is larger. At 3 s^–1^, chains start to stretch and birefringence is detected without the λ/2 plate while no birefringence can be detected with the λ/2 plate, since at this shear rate the stress-optical rule still works and the birefringence is intrinsic. Similarly at 10 s^–1^, stronger birefringence is detected without the λ/2 plate while no birefringence can be detected with the λ/2 plate. At 45 s^–1^, where both the birefringence and C have jumped up as was shown in Figures 4 and 5a, the stress-optical rule fails and now birefringence can be detected both with and without the λ/2 plate. Hence, in the nematic regime above 45 s^–1^ HDPE exhibits form birefringence. Form birefringence likely indicates bundling of stretched chains. Whether this bundling creates a shear band spanning the sample or just creates shish cannot be determined from our methods.

## Discussion

### Polymer Phase Map

At a particular sample thickness, the transmitted path difference can provide information regarding when the sample transitions first from an isotropic melt to a biphase and then to the nematic regime where a large fraction of long chains are nematic. This provides a nice way to determine these two transitions at any temperature, just based on one SIPLI experiment that captures the birefringence at all shear rates lower than the perimeter shear rate. Hence, upon repeating the shearing experiments at higher temperatures, 131, 135, and 139 °C, a phase map for HDPE can be constructed^60^ as a function of shear rate as shown in Figure 7a. In the isotropic regime the melt exhibits no measurable birefringence (Figure 3) and the Cox–Merz rule works well. Entering the biphase regime is when the Cox–Merz rule first fails and birefringence is strongly detected (white light source). In the nematic regime, the sample exhibits a shear viscosity scaling as the reciprocal square root of shear rate which acts as evidence of flow-induced nematic alignment of long chains.

**Figure 7:**
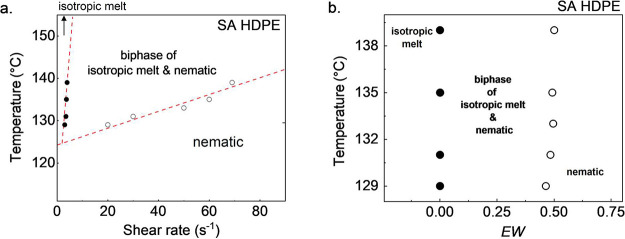
Phase map for HDPE as a function of (a) shear rate and (b) the control variable for nematic alignment *EW*, where *E* is the fraction of stretched chains and *W* is the specific work required to reach a steady nematic viscosity for the three temperatures above Strobl’s zero growth temperature of 132.5 °C. The 60 and 69 s^–1^ experiments at 129 and 131 °C, respectively, did not show a nematic steady viscosity and W was taken where the normal stress starts to upturn, indicating that the crystal precursor network started to form from the nematic alignment. The closed symbols represent where the melt first transitions from an isotropic phase to a biphase of isotropic and nematic chains, manifested by a sudden failure of the Cox–Merz rule and strong birefringence. The open symbols represent the transition from the biphase regime to the nematic regime, where shear viscosity starts scaling as the reciprocal square root of shear rate which acts as evidence of flow-induced nematic alignment of long chains. The *EW* values for the closed and open symbols are 2 × 10^–4^ and 0.5, respectively, independent of temperature.

In Figure 7b the phase map is recast in terms of *EW* using eq 4, where *E* is the fraction of stretched chains and *W* is the specific work required to reach a steady nematic state at the respective shear rates during a start-up of steady shear experiment. The *EW* value for the closed and open symbols is 2 × 10^–4^ and 0.5, independent of temperature, suggesting that *EW* is the critical parameter for flow-induced nematic alignment. The flow curve was also constructed at 139 °C to plot the transition shear rates in the phase map, showing that the birefringence method used to determine transition shear rates works well at 131 and 135 °C as they fall between the transition shear rates at 129 and 139 °C. The isotropic-to-nematic transition shear rate increases with increasing temperature,^61,66^ as expected. The biphase region widens as temperature is increased.

This biphase region has the characteristics of a shear band^63,64^ wherein isotropic chains and nematic chains coexist. In this scenario, there are two horizontal bands that have the same viscosity, one containing stretched long chains and the other isotropic shorter chains that orient somewhat but are not stretched. Suspensions of rigid-rod-like particles that exhibit banding in shear show a similar widening of the band^65^ as concentration is lowered (lower concentration is like higher temperature).

As the shear rate is increased, more chains are stretched and the band with the stretched long chains grows and the melt eventually transitions to the nematic regime (open symbols in Figure 7). In the nematic regime the shear stress and birefringence start to scale as the square root of shear rate. Eventually at higher shear rates in the nematic regime the horizontal shear band spans across the width of the sample (but is not the entire sample) and this is when form birefringence appears as denoted by the jump in Δ*n* in Figure 4 and the jump in C in Figure 5a. Form birefringence also occurs at 145 °C where no crystallization can occur,^66^ further proving that the form birefringence is from nematic alignment only. The intercept of the biphase-to-nematic line on the *y*-axis in Figure 7a estimates the equilibrium isotropic to nematic transition at 124 °C if the polymer did not crystallize instead.

### Nematic Alignment in Polymer Melts

PEEK, poly­(sulfone) (PSU) and HDPE all show a nematic alignment of chains in sufficiently strong shear flow. It is important to point out that isotactic polypropylene (iPP) never shows any evidence of nematic alignment. There is no strong birefringence in the SIPLI for iPP in shear^2,3,5,6,9^ and it shows a power law shear thinning exponent^2^ of −0.7, typical of polydisperse entangled linear polymers. There are two differences between these polymers that are worth discussing.

PEEK, PSU and HDPE all have Kuhn monomers with very high aspect ratio, by virtue of having no side groups. In contrast, iPP has a methyl group attached to every other carbon in the backbone, greatly lowering the aspect ratio of the iPP Kuhn monomer, which might make iPP less prone to nematic alignment. In the capillary rheometer at very high shear rates, iPP never shows the Marrucci shear thinning slope of −1/2 that is characteristic of nematic alignment of chains. However, iPP does crystallize into the shish-kebab morphology after the interval of shear at those very high rates. A summary of Kuhn monomer aspect ratios and estimated Kuhn monomer excluded volume for some commonly studied polymers is presented in Table 1. The horizontal gap separates iPP and polystyrene (PS) that never achieve nematic alignment from PEO, HDPE, PSU and PEEK that do attain nematic alignment at reasonable shear rates. Syndiotactic polypropylene (s-PP) might be capable of forming a nematic as opposed to iPP. s-PP^22^ has $b^{2}d/v_{k}$ = 2.8 which is close to the $b^{2}d/v_{k}$ = 2.9 of HDPE. Other notable mentions include s-PMMA.^22^ Atactic-PMMA has $b^{2}d/v_{k}$ = 2 which likely will not exhibit nematic alignment, while s-PMMA has $b^{2}d/v_{k}$ = 3. In the future it would be interesting to explore this effect of tacticity on nematic alignment in polymers. Other common atactic polymers like poly­(vinyl methyl ether) (PVME) and poly­(vinyl acetate) (PVAc),^22^ have $b^{2}d/v_{k}$ of 2.2 and 2.3, respectively suggesting they might be interesting candidates for future study, to see whether they are capable of exhibiting nematic alignment.

**Table 1:** Kuhn Monomer Aspect Ratios and Excluded Volumes for Some Common Polymers

	Kuhn length, *b*	Kuhn monomer volume, *v*_*k*_	overlap parameter of Kuhn monomers, $\frac{b^{3}}{v_{k}}$	cylindrical Kuhn monomer diameter, *d* = $\sqrt{\frac{v_{k}}{b}}$	cylindrical Kuhn monomer excluded volume, *b* ^2^ *d*	$\frac{b^{2}d}{v_{k}}$ ^a^
polymer	(nm)	(nm^3^)		(nm)	(nm^3^)	
iPP	1.1	0.41	3.7	0.60	0.78	1.9
PS	1.8	1.24	4.5	0.84	2.65	2.1
						
PEO	1.1	0.21	6.3	0.44	0.53	2.5
HDPE	1.4	0.32	8.1	0.48	0.91	2.9
PSU^60^	6.6	3.12	92	0.69	30	9.6
PEEK^4,67^	10.8	2.50	500	0.48	56	22

a *b* ^2^ *d* is proportional to the excluded volume of a Kuhn monomer (assuming a cylinder),^16^ making $\frac{b^{2}d}{v_{k}}$ an overlap parameter^68^ for excluded volume.

Another consequence of having no side groups is that PEEK and HDPE both adopt a highly aligned conformation^4,26,29,69^ when they crystallize. PEEK chooses a straight-chain crystal form that maximizes Π-stacking^70^ and the lowest energy crystal form of HDPE^71^ has the chains in a planar all-trans zigzag conformation. This all-trans conformation loses nearly all configurational entropy and is the lowest energy conformation;^72^ once formed this ground state should be quite stable. At 129 °C using shear rates of up to 120 s^–1^, there is a Raman peak at 1129 cm^–1^ because of strong nematic alignment (see Supplementary Figure 5 for the Raman spectra at each shear rate). iPP is radically different; the methyl groups raise the energy of the all-trans conformation^73^ and the crystal forms of iPP all have conformations that are sequenced mixtures of trans and gauche that allow iPP to form a helical conformation^74^ that then can crystallize. There is simply no reason to expect shear flow to be able to force iPP into the necessary sequence of trans and gauche needed to create the helical conformation.

## Experimental Section

### Methods

#### Rheology

In our rheology experiments, strain-controlled rotational rheometers ARES-G2 (TA Instruments, New Castle, DE) and ARES-LS (Rheometric Scientific) are equipped with cone and plate and cone-partitioned plate (CPP) fixtures to impose shear, and monitor the linear viscoelastic oscillatory response of polymers. CPP is a unique geometry that helps measure reliable data even when edge fracture occurs^75^ near the perimeter. In both cone and plate and CPP, there is a constant shear rate γ̇ across the radius of the puck where the torque is measured.

#### Shear Induced Polarized Light Imaging (SIPLI)

Mykhaylyk and colleagues^8,24,25^ developed the shear-induced polarized-light imaging (SIPLI) setup. Rheo-optical detection in polymers dates back many decades,^53,76^ where flow birefringence was studied in polymer solutions and melts using optical signatures to infer chain orientation and stress. SIPLI helps extend early rheo-optical work for polymer melts and complex fluids in a seamless manner, as SIPLI is compatible with standard rheometer geometries such as parallel-plate and cone–plate. SIPLI entails a stress-controlled rotational rheometer (Anton Paar MCR-502) equipped with parallel circular plates where there is a distribution of the γ̇ and *W* across the radius of the puck, with the highest γ̇ being at the perimeter; and both γ̇ and *W* zero at the center. The bottom plate has no rheometric function and is replaced with transparent quartz. The top plate is precisely 25 mm in diameter and polished to reflect. The transparent plate provides the optical access path for light, which is reflected off the polished top plate. The SugarCUBE (Edmund Optics, Barrington, NJ) is used to transmit the light intensity through a fiber optic which is then aligned with a camera (CCD) to ensure even field illumination across the plate. Samples are loaded in the gap between plates where a specific gap height (d) is chosen to balance optical signal, as retardation is proportional to sample thickness (= twice the gap height). Optical signal is balanced by using a larger thickness for higher order colors and a smaller thickness for lower order colors, so that all data fall within the Michel-Levy chart at different locations to ensure the retardation method is accurate to determine birefringence. The incident light passes through a single polarizer, through the sample twice and then through an analyzer (perpendicular to the polarizer) for rapid monitoring of birefringence. Our CCD camera (Lumenera Lu165C color CCD camera) has a frame rate of 15 frames per second. The polarizer and analyzer being perpendicular to one another form a Maltese cross (extinction) as shown in Figures 3 and 4, that always remains dark even when birefringence is detected. The 2D SIPLI images captured during experiments are stacked in an imaging software called FIJI (commonly referred to as ImageJ). A slice analysis is then conducted at 45° on the stack to attain time- and shear rate resolved birefringence data.

#### In situ Raman during Rheology

The in situ Rheo-Raman data were collected with the help of a loaner instrument, Cora 5001 Raman Spectrometer, from Anton Paar Co., Ltd., Graz, Austria. A Raman probe (λ = 785 nm) was set up at a radial position corresponding to a specific shear rate in the SIPLI. A new sample was used for each shear rate. The Raman spectra were collected using a 30 s exposure time and the laser power was set to 450 mW.

## Supplementary Material













## Data Availability

All data are included in the article and/or Supporting Information.

## Notes

Molecular weight distribution input into BoB; rheological master curve frequency scale shift; relaxation times of HDPE; entanglement relaxation time from LVE and BoB; predicted number of Kuhn monomers; 2D-SIPLI image of HDPE using a 633 nm wavelength laser; red light intensity at perimeter shear rates of 5, 15, and 40 s^–1^ using the slice analysis; Raman spectra of sheared HDPE at 129 °C; and references (PDF)
All data are included in this excel file­(XLSX)
HDPE nematic at 129 °C using a perimeter shear of 45 s^–1^ (1× speed)­(MP4)
HDPE nematic at 129 °C using a perimeter shear of 45 s^–1^ (0.25× speed)­(MP4)
HDPE nematic at 129 °C using a perimeter shear of 75 s^–1^ (1× speed)­(MP4)
HDPE nematic at 129 °C using a perimeter shear of 75 s^–1^ (0.25× speed)­(MP4)

## Abbreviations

(HDPE): high-density polyethylene
(E): energy conversion factor
(W): specific work
