# Supplementary material for: When Flow Creates Order: Nematic Alignment and Form Birefringence in Shear for High-Density Polyethylene Melts
Source: Macromolecules. 2026 Jul 20;59(15):8750–9. doi: 10.1021/acs.macromol.6c00994 (PMC13472172; doi:10.1021/acs.macromol.6c00994)
Supplement: Supplementary file 1 [file ma6c00994_si_001.pdf]

Supporting Information for When flow creates order: Nematic alignment and form birefringence in shear for high-density polyethylene melts

Arshiya Bhadu[1], Alicyn M. Rhoades*[2], and Ralph H. Colby*[1]

[1]Department of Materials Science and Engineering, Penn State University, University Park, PA 16802, United States
[2]School of Engineering, Penn State Behrend, Erie, PA 16563, United States

*E-mail: amh234@psu.edu, rhc@plmsc.psu.edu

Characterization of molecular weight distribution of HDPE

To quantify the concentrations of the longest chains in HDPE, its molecular weight distribution needs to be measured. The gel permeation chromatography (GPC) experiment of HDPE was performed on an Agilent 1260 Infinity Multi-Detector at 160°C equipped with Agilent 1260 Infinity II HT-ELSD (G7826A). HPLC grade 1,2,4-Trichlorobenzene (TCB) is used as the eluent with 0.015 percent BHT for stabilization. First, 5-10 mg of the polymer pellets are dissolved in 1,2,4-Trichlorobenzene (TCB) to obtain a concentration of 1.5-2 mg mL^{-1}. The dn/dc of HDPE in TCB[1] is 0.109 mL mg^{-1}. To ensure complete dissolution, samples were kept at 160°C for 90 min. Polymer solutions (200 µL) are then injected at a sampling interval of 1 s for an 11 min run with a flow rate of 1 mL min^{-1}. Using polystyrene (PS) and polyethylene (PE) narrow standards for the calibration curve, the number-average molecular weight (M_n), weight-average molecular weight (M_w), and dispersity (M_w/M_n) of the HDPE were determined using the Agilent GPC / SEC 2.2 software. The molar mass of the samples was calculated using the reported Mark–Houwink parameters[2] $K_{PS} = 23.2 \times 10^{-5}$ cm^3 g^{-1}, a_{PS} =0.653 , $K_{PE} = 51.7 \times 10^{-5}$ cm^3 g^{-1} , and $a_{PE} = 0.706$. The HDPE sample has $M_w = 102.5$ kg mol^{-1} and a dispersity $M_w/M_n = 10.4$.

Estimation of relaxation times of long chains in HDPE using BoB

For monodisperse and polydisperse linear chains, BoB[3] takes as input the number of Kuhn monomers in an entanglement strand (N_e), entanglement strand Rouse time (τ_e), monomer molecular weight, melt density, and the molecular weight distribution (as derived from GPC). The molecular weight distribution is input to BoB as a list of monodisperse components with corresponding weight fractions from the GPC data assuming all chains are linear (no long chain branching). Supplementary Table 1 shows the respective weight fraction of linear chains input into BoB. BoB is then used to calculate the linear viscoelastic response (LVE) of the HDPE sample. This is done using Wolfram Mathematica[3,4] to fit the monodisperse components (59 linear chain molecular weights shown in Supplementary Table 1) to the shape of the cumulative weight fraction versus log molecular weight. BoB determines the LVE response of the polymer. This is then compared to the LVE response of HDPE using a rheometer in Supplementary Figure 1. The rheological measurements were done using a strain-controlled ARES G2 rotational rheometer (TA Instruments, New Castle, DE), and the experiments were run under nitrogen to minimize sample oxidation. The rheometer was equipped with 25 mm Cone and Plate geometry to perform frequency sweeps at 12 temperatures (see Supplementary Table 2) with frequencies ranging from 0.001 to 628 rad s^{-1}. A strain amplitude of 0.01 was used, while at lower frequencies, the strain amplitudes were increased to provide sufficient torque levels while remaining in the linear viscoelastic regime. Using a_T, the activation energy for flow, irrespective of temperature, is determined to be $E_a = 32$ kJ mol^{-1} from the slope of ln (a_T) vs $1/T$. This E_a compares well with literature values[5] for linear polyethylenes.

Supplementary Table 1: Molecular weight distribution input into BoB.

Weight fraction, w_N	M (kg mol^{-1})
0.00832	0.368
0.00916	0.709
0.00977	1.088
0.01105	1.502
0.01233	1.950
0.0131	2.426
0.0141	2.931
0.01456	3.462
0.01535	4.018
0.01627	4.612
0.0173	5.250
0.01765	5.927
0.01823	6.636
0.01838	7.382
0.01915	8.172
0.01961	9.022
0.02059	9.942
0.02071	10.935
0.02102	11.993
0.02167	13.139
0.01932	15.593
0.02098	16.908
0.0201	18.349
0.02037	19.889
0.01902	21.520
0.02029	23.301
0.01977	25.301
0.02012	27.514
0.02088	30.0612
0.01876	32.841
0.0194	35.866
0.01922	39.339
0.01894	43.241
0.01871	47.617
0.01851	52.518
0.01816	57.968
0.01799	64.000
0.01802	70.722
0.01783	78.185
0.01789	86.469
0.01794	95.714
0.01785	106.034
0.01787	117.574
0.01776	130.563
0.01775	145.270
0.01762	162.063
0.01755	181.441
0.01722	204.003
0.01701	230.465
0.01676	262.140
0.01628	300.804
0.01561	348.644
0.0149	409.225
0.01405	488.438
0.01299	597.265
0.0118	759.390
0.01022	1033.130
0.00812	1589.146
0.00518	2040.749

Supplementary Table 2: Rheological master curve frequency scale shift factors[6] for HDPE at $T_{ref} = 145$°C.

Temperature (°C)	a_T ($T_{ref} = 145$°C)
125	1.445
129	1.468
130	1.370
132	1.356
133	1.383
140	1.107
145	1
170	0.577
190	0.376
210	0.309

Supplementary Figure 1 compares the LVE curve of HDPE from the rheometer as open symbols versus the LVE curve from BoB as solid lines; (a) T = 145°C, (b) T = 135°C, (c) T = 129°C. The entanglement molecular weight of HDPE has been reported[7,8] to be $M_e = 1040$ g mol^{-1}. The number of Kuhn monomers in an entanglement strand was calculated by dividing M_e by the Kuhn monomer molecular weight[7,8] ($M_o =$ 150 g mol^{-1}) and $N_e = 6.9$. The other values input into BoB were the mass density (0.853 g cm^{-3} at 25°C) and the temperature. The entanglement strand Rouse time (τ_e) was determined by comparing the LVE-response of HDPE and the curves from BoB modeling. The Rouse time of a Kuhn monomer $\tau_0 = \tau_e/Ne^2$ and τ_e are reported in Supplementary Table 3.

Supplementary Table 3: Relaxation times of HDPE used in this study at temperatures ranging from 145°C to 129°C.

T (°C)	τ_o (ns)	τ_e (ns)
129	0.134	6.40
131	0.128	6.10
133	0.122	5.83
135	0.117	5.56
139	0.104	4.97
145	0.095	4.50

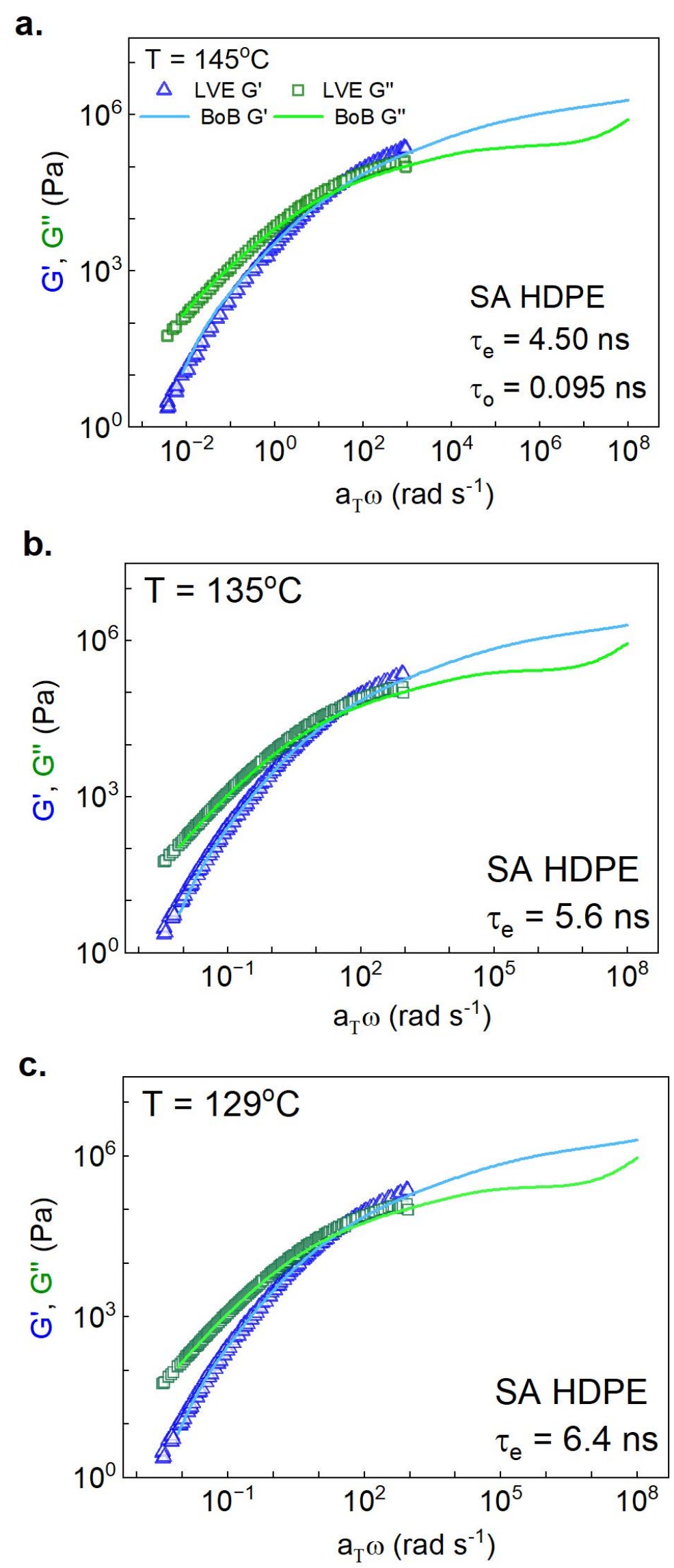

Supplementary Figure 1: Entanglement relaxation time of from LVE and BoB. Comparison at (a) 145°C, (b) 135°C, and (c) 129°C of the LVE master curve of HDPE from time-Temperature superposition (open symbols) with the LVE curve from BoB analysis (solid curves) of the molecular weight distribution of HDPE.

N_{str} as a function of shear rate using the E model

Using τ_o from BoB, the number of Kuhn monomers in the shortest chains that get stretched at a given shear rate N_{str} can be determined at any temperature as a function of shear rate, as shown in Supplementary Figure 2 at 129°C.

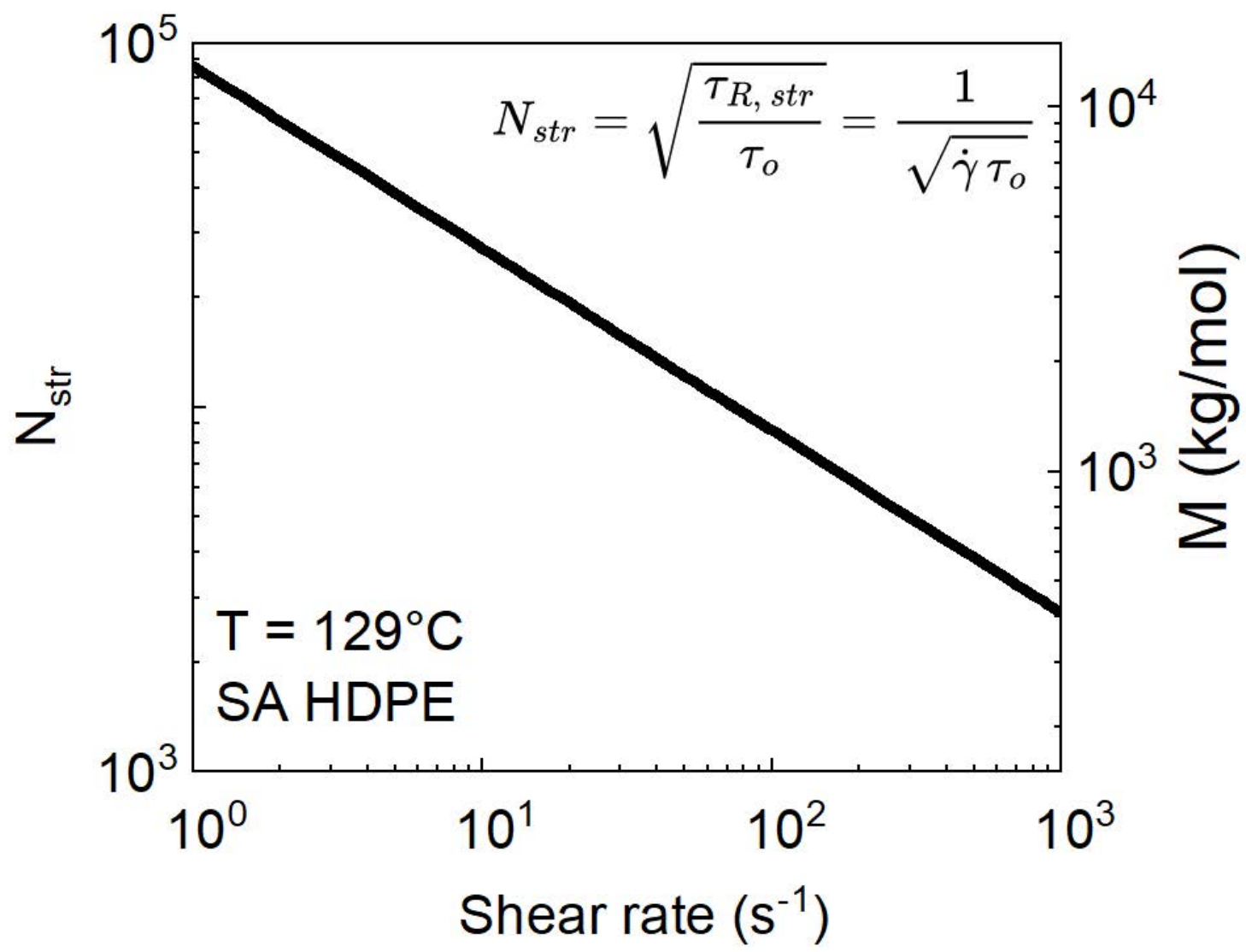

Supplementary Figure 2: The predicted number of Kuhn monomers in the shortest chain that gets stretched N_{str}, at 129°C, as a function of shear rate using the Rouse time of the Kuhn monomer $\tau_o = 0.134$ ns. As shear rate increases, N_{str} decreases, meaning more chains get stretched. The right axis shows the corresponding molecular weight $M_{str} = M_o N_{str}$

Calibration of light intensity

Δn at 40 s^{-1} was used to plot the light intensity in terms of birefringence in Figure 2 in the paper, where the light intensity was measured using a white light source equipped with a filter for 633 nm wavelength light (red light). The red light intensity as a function of radius (corresponding to a specific shear rate) was calculated using FIJI along a slice of the 2D image during shear (see Supplementary Figure 3).

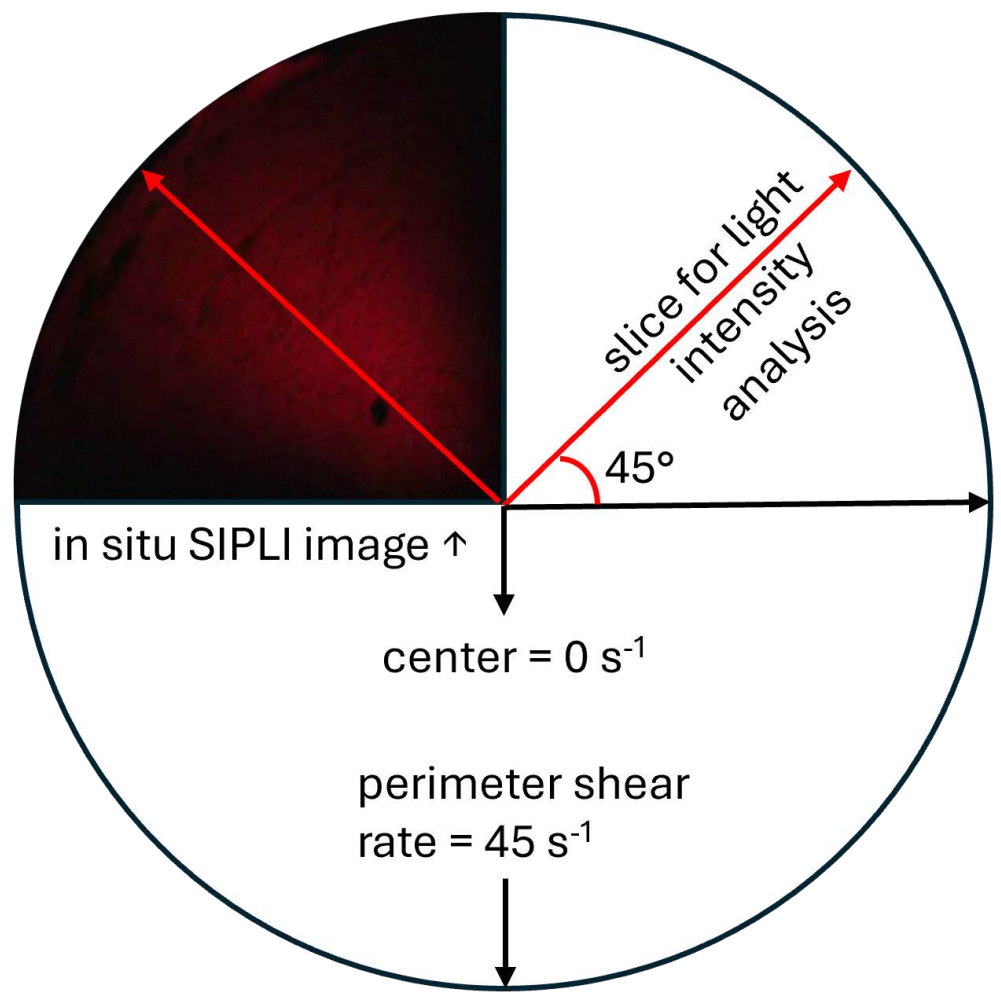

Supplementary Figure 3: Light intensity during shear of HDPE using a 633 nm wavelength laser at a shear rate of 45 s^{-1}. The slice corresponds to the line that was used to calculate light intensity as a function of shear rate at a 45° angle from the Maltese cross. A monochromatic source of light allows for better quantification of light intensity as it only measures changes at a single wavelength.

Similarly, the red light intensity at 5 and 15 s^{-1} were also evaluated using lower light intensity settings to avoid light saturation and to accurately capture the shear rate dependence of birefringence as shown in Supplementary Figure 4. The noise in the data is a result of surface defects on the polished reflecting top plate in the SIPLI.

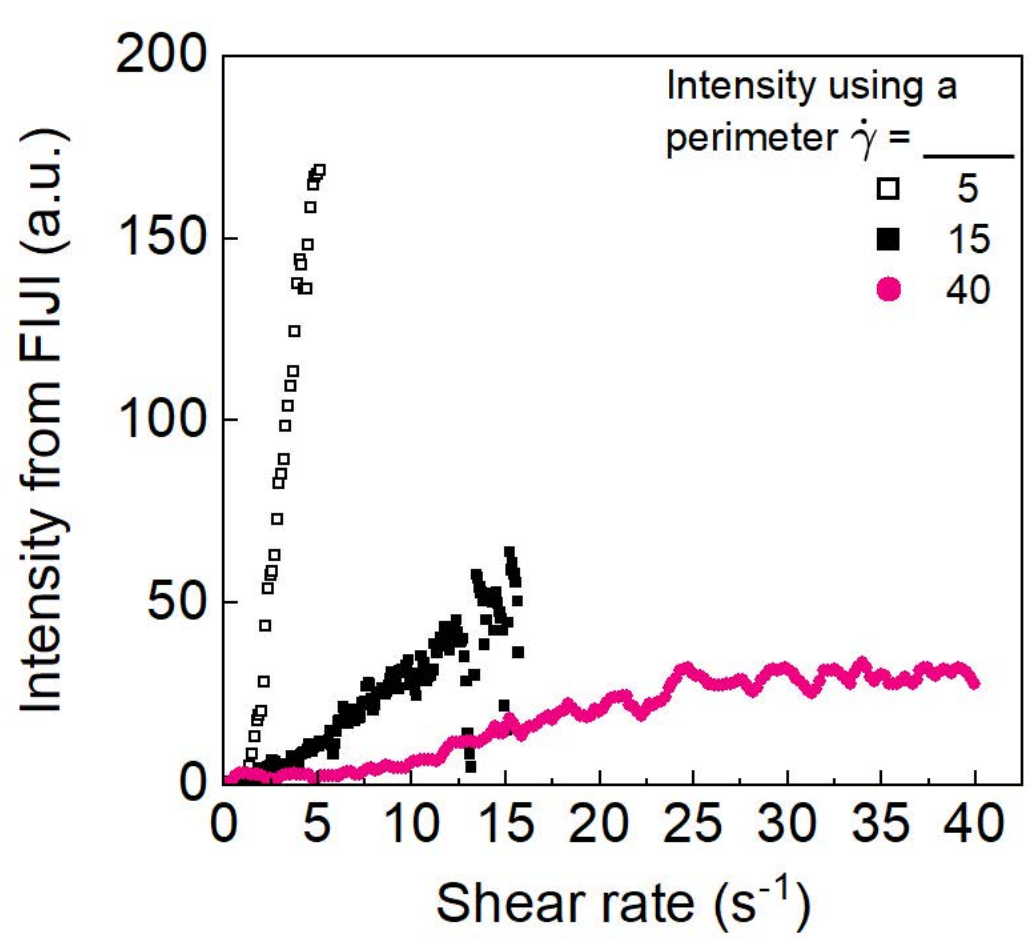

Supplementary Figure 4: Light intensity, using a 633 nm wavelength of red light, from the slice analysis method shown in Supplementary figure 3 during shear of HDPE at various perimeter shear rates of 5, 15, and 40 s^{-1}. These intensity values were then calibrated using the birefringence values at 11.7 and 40 s^{-1} determined using the path difference from the Michel-Lévy chart[9] for a thicker sample.

Raman Intensity under shear

At 129°C, using various shear rates, there is a peak at 1129 cm^{-1}, as shown in Supplementary Figure 5. The Raman intensity curves were shifted for clarity in Figure 2d of the main paper, but are shown here without any normalization or shifting. The raw Raman intensity at 1129 cm^{-1} is then what is plotted in Figure 2d of the main paper to compare to the E model.

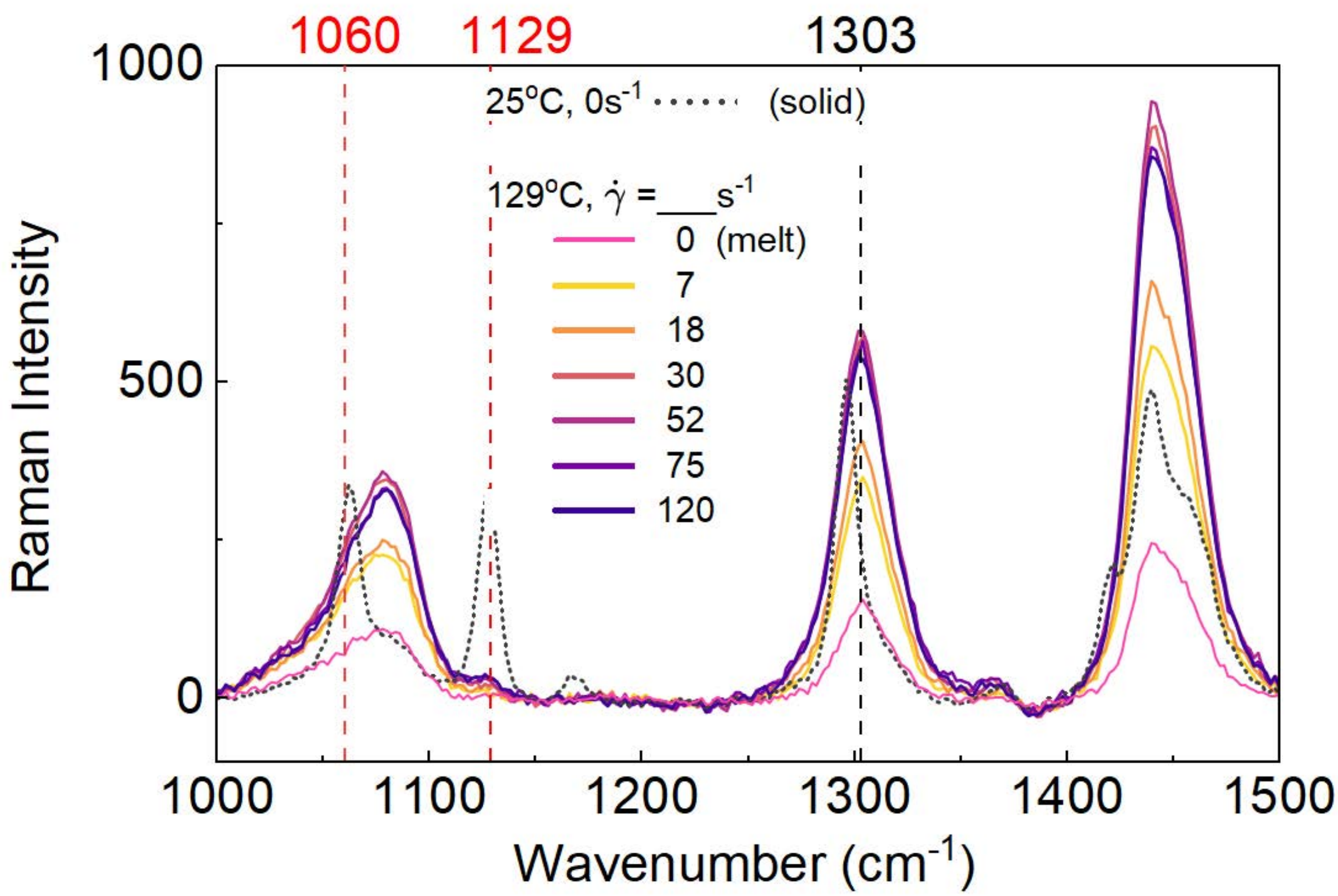

Supplementary Figure 5: Raman spectra of sheared HDPE at 129°C. HDPE was sheared at 129°C (near its melting point of 129.5^{o}C) using various shear rates, where a peak was detected at 1129 cm^{-1} at all shear rates (largest at the highest shear rate) and was absent in the isotropic melt at 0 s^{-1}. The 1129 cm^{-1} represents a conformation with more than ten consecutive trans ethylene repeat units,[10–13] which is generally only otherwise observed in a solid semi-crystalline HDPE (dotted curve). The rotator phase of HDPE shows a peak at 1296 cm^{-1}, which is not observed in the nematic phase.

Caption for Movie S1. HDPE nematic at 129°C using a perimeter shear of 45 s^{-1} (1x speed). The video is an animated version of one of the shearing experiments in Figure 3 of main manuscript. HDPE was sheared in the SIPLI at 129°C using a perimeter shear of 45 s^{-1} at 1.4 mm sample thickness. The speed of the video is in real time (1x speed). The birefringence increases as a function of radius or shear rate.

Caption for Movie S2. HDPE nematic at 129°C using a perimeter shear of 45 s^{-1} (0.25x speed). The video is an animated version of one of the shearing experiments in Figure 3 of main manuscript. HDPE was sheared in the SIPLI at 129°C using a perimeter shear of 45 s^{-1} at 1.4 mm sample thickness. The speed of the video is in 1/4th of real time (0.25x speed) so that the video can be followed better. The birefringence increases as a function of radius or shear rate.

Caption for Movie S3. HDPE nematic at 129°C using a perimeter shear of 75 s^{-1} (1x speed). The video is an animated version of one of the shearing experiments in Figure 3 of main manuscript. The video of one of the shearing experiments in Figure 3 of main manuscript. HDPE was sheared in the SIPLI at 129°C using a perimeter shear of 75 s^{-1} at 1.4 mm sample thickness. The speed of the video is in real time (1x speed). The birefringence increases as a function of radius or shear rate.

Caption for Movie S4. HDPE nematic at 129°C using a perimeter shear of 75 s^{-1} (0.25x speed). The video is an animated version of one of the shearing experiments in Figure 3 of main manuscript. The video of one of the shearing experiments in Figure 3 of main manuscript. HDPE was sheared in the SIPLI at 129°C using a perimeter shear of 75 s^{-1} at 1.4 mm sample thickness. The speed of the video is in 1/4th of real time (0.25x speed) so that the video can be followed better. The birefringence increases as a function of radius or shear rate.

References

[1] Pope, J. W.; Chu, B. A laser light scattering study on molecular weight distribution of linear polyethylene. *Macromolecules* **1984**, *17*, 2633, DOI: 10.1021/ma00142a031.

[2] Mark, J. E. *Physical Properties of Polymers Handbook*; Springer, 2007.

[3] Das, C.; Inkson, N. J.; Read, D. J.; Kelmanson, M. A.; McLeish, T. C. B. Computational linear rheology of general branch-on-branch polymers. *J. Rheol.* **2006**, *50*, 207–234, DOI: 10.1122/1.2167487.

[4] Hamad, F. G.; Colby, R. H.; Milner, S. T. Onset of Flow-Induced Crystallization Kinetics of Highly Isotactic Polypropylene. *Macromolecules* **2015**, *48*, 3725–3738, DOI: 10.1021/acs.macromol.5b00386.

[5] Gahleitner, M. Melt rheology of polyolefins. *Prog. Polym. Sci.* **2001**, *26*, 895–944, DOI: 10.1016/S0079-6700(01)00011-9.

[6] Dealy, J. M.; Wissbrun, K. F. *Melt Rheology and Its Role in Plastics Processing: Theory and Applications*; Springer Science & Business Media, 2012.

[7] Rubinstein, M.; Colby, R. H. *Polymer Physics*; Oxford University Press, 2003; DOI: 10.1093/oso/9780198520597.001.0001.

[8] Fetters, L. J.; Lohse, D. J.; Colby, R. H. In *Physical Properties of Polymers Handbook*; Mark, J. E., Ed.; Springer, 2007; pp 447–454, DOI: 10.1007/978-0-387-69002-5_25.

[9] Bloss, F. D. *Optical Crystallography (Monograph Series)*; Mineralogical Society of America, 1999.

[10] Meier, R. J. Studying the length of *trans* conformational sequences in polyethylene using Raman spectroscopy: a computational study. *Polymer* **2002**, *43*, 517–522, DOI: 10.1016/S0032-3861(01)00416-5.

[11] Kotula, A. P.; Meyer, M. W.; Vito, F. D.; Plog, J.; Walker, A. R. H.; Migler, K. B. The rheo-Raman microscope: Simultaneous chemical, conformational, mechanical, and microstructural measures of soft materials. *Rev. Sci. Instrum.* **2016**, *87*, 105105, DOI: 10.1063/1.4963746.

[12] Tasumi, M.; Shimanouchi, T.; Miyazawa, T. Normal vibrations and force constants of polymethylene chain. *J. Mol. Spectrosc.* **1962**, *9*, 261–287, DOI: 10.1016/0022-2852(62)90236-9.

[13] Kida, T.; Janchai, K.; Tokumitsu, K.; Yamaguchi, M. Flow-induced crystallization behavior of high-density polyethylene evaluated by Rheo-Raman spectroscopic system. *Polym. J.* **2023**, *55*, 1141–1150, DOI: 10.1038/s41428-023-00821-5.
